# Survival of Patients With UrAC and Primary BAC and Urothelial Carcinoma With Glandular Differentiation

**DOI:** 10.3389/fonc.2022.860133

**Published:** 2022-05-12

**Authors:** Tao Wang, Zheng Lv, Huayi Feng, Jinlong Li, Bo Cui, Yang Yang, Xing Huang, Xiangyi Zhang, Xintao Li, Xin Ma

**Affiliations:** ^1^ Medical School of Chinese PLA, Beijing, China; ^2^ Department of Urology, The Third Medical Centre, Chinese PLA General Hospital, Beijing, China; ^3^ Department of Urology, The Tianjin Third Central Hospital Affiliated of Nankai University, Beijing, China; ^4^ Department of Pathology, The First Medical Centre, Chinese PLA General Hospital, Beijing, China; ^5^ Department of Urology, Air Force Specialty Medical Center, Beijing, China

**Keywords:** urachal adenocarcinomas, primary bladder adenocarcinomas, urothelial carcinoma with glandular differentiation, overall survival, mucinous adenocarcinoma, multivariate analysis

## Abstract

**Purpose:**

To investigate the significance of demographic and pathological characteristics on the survival outcomes of urachal adenocarcinoma (UrAC), primary bladder adenocarcinoma (BAC) and urothelial carcinoma with glandular differentiation (UCGD) in China.

**Materials and Methods:**

We retrospectively analyzed cases with non-distant metastases (≤ T4M0). Of 106 patients, 30 (28.3%), 40 (37.7%), and 36 (34.0%) met the criteria for UrAC, primary BAC, and UCGD, respectively. Data on patient demographics, tumor pathology, and survival outcomes were collected. The median follow-up was 36 months. Survival was analyzed using multivariate Cox regression.

**Results:**

Patients with UrAC were younger (51.87 ± 15.25 years) than those with primary BAC (60.50 ± 12.56 years) and UCGD (63.83 ± 11.60 years) (*P*<0.001). Patients with UrAC were the most likely to be stage T3–4 (70.0% *vs*. 40.0% *vs*. 44.4%; *P*<0.001), while the primary BAC group had a higher rate of poor differentiation than the UrAC and UCGD groups (57.4% *vs*. 18.5% *vs*. 24.1%; *P*<0.001). The Kaplan–Meier curves showed that the overall survival (OS), progression-free survival (PFS), and disease-specific survival (DSS) of the primary BAC group were poorer than those of both the UrAC and UCGD groups (P=0.0046,P<0.0001,P=0.0077 respectively). Regarding BAC, patients with mucinous adenocarcinoma tended to have better OS and PFS than those with other histological types (P<0.005,P=0.0245). Multivariate Cox regression analysis revealed that tumor type (*P*=0.002), T stage (*P*=0.034), and the age-adjusted Charlson Comorbidity Index (aCCI) scores (*P*=0.005) predicted the postoperative OS and DSS of the patients. For PFS, the tumor type (*P*=0.011), grade (*P*=0.000), and aCCI (*P*=0.002) scores were predictive.

**Conclusion:**

Among UrAC, primary BAC, and UCGD patients, the prognosis was poorest for those with primary BAC. Attempts should be made to diagnose these aggressive tumors early, since patients in whom tumors are detected early appear to survive longer.

## Introduction

Bladder adenocarcinoma (BAC) is rare, accounting for only 0.5–2% of bladder malignancies (1, 2). According to clinical and pathology multidisciplinary diagnostic criteria, BAC can be classified as urachal adenocarcinoma (UrAC), primary BAC, and metastatic adenocarcinoma. UrAC accounts for about 10% of BAC (3, 4). The incidence of BAC is higher in patients with bladder exstrophy (90%) and in regions where schistosomiasis is endemic (9%–10%) (5), owing to the rarity of the disease, the pathogenesis and natural history of BAC have not been well defined (6).

Primary BAC is usually treated with radical cystectomy, while UrAC is usually treated with partial cystectomy; thus, differentiating these two diseases is very important (7). There is good reason to believe that the prognoses of UrAC and primary BAC differ (8). In most studies, UrAC had a better prognosis than primary BAC (8–11). Most of these studies examined the Surveillance, Epidemiology, and End Results (SEER) database, which were limited by race, economy, education level, medical technology development level and incomplete data although the cases were large (8, 9). While these data are of great significance to the American population, China lacks long-term, reliable data for prognostic evaluation of UrAC and primary BAC, as a country with a high incidence of bladder cancer.

Urothelial carcinoma (UC) is the most common histological type of bladder cancer and is characterized by histological variation. The 2016 World Health Organization (WHO) classification of urothelial and reproductive system tumors distinguishes 12 subtypes, the prognoses of which differ significantly from that of typical UC, usually characterized by high grade and stage on discovery, rapid progression, and high recurrence and metastasis rates (12). Glandular differentiation, the second most common subtype, accounts for about 6% of UCs and is diagnosed based on true glandular involvement, excluding pseudoadenoid areas caused by necrosis and cells containing intracellular mucus (8). Many studies have reported prognosis differences between urothelial carcinoma with glandular differentiation (UCGD) and UC, UC and BAC. However, the prognosis difference between BAC and UCGD, two clinically differentiated diseases, is rarely reported.

Therefore, this study compared the prognosis and prognostic factors of three pathological types of bladder tumor: UrAC, primary BAC, and UCGD. Our results may provide guidance for Chinese clinicians.

## Materials And Methods

### Population and Follow-Up

All patients were diagnosed based on clinicopathological findings. To increase comparability, we enrolled only patients with non-distant metastases (≤ T4M0), including 30 patients with UrAC, 40 with primary BAC, and 36 with UCGD, diagnosed and treated in the Urology Department of the Chinese People’s Liberation Army General Hospital from 2005 to 2019. Patient characteristics were obtained through telephone consultations and inpatient medical records.

• We used the following diagnostic criteria for UrAC, as revised by Gopalan A et al. (13): Location of the tumor in the bladder dome and/or anterior wall; Epicentre of carcinoma in the bladder wall; Absence of widespread cystitis cystica and/or cystitis glandularis beyond the dome or anterior wall; Absence of a known primary elsewhere. A diagnosis of primary BAC should be considered after the exclusion of UrAC and metastatic adenocarcinoma. We added typical histological images of three tumors (**Supplementary Figure 2**). Pathological staging was based on the TNM staging criteria of the American Joint Committee on Cancer (AJCC) (8^th^ edition, 2017) to cover all three masses simultaneously. Due to the relatively small sample size, BAC was subdivided into three histological subtypes: mucinous, not otherwise specified (NOS), and others. Considering that there is currently no recognized histological grading system for UrAC, we adopted the method of Pinthus et al. (14) from well through moderately to poorly differentiated tumors. We divided KI67 into gradient groups, that is, less than or equal to 50, greater than 50 and undetermined. According to Hjalmarsson et al. (15), an age-adjusted Charlson Comorbidity Index (aCCI) of 0–2, 3–5, and 6–8 indicates low, medium, and high risk, respectively. Body mass index (BMI) calculations were based on Asian criteria.

### Statistical Analysis

Follow-up tables were constructed for all 106 patients based on baseline information, survival time, and survival status data. Overall survival (OS) was defined as the time from first treatment to patient death or the study endpoint. Progression-free survival (PFS) was defined as the time from first treatment to tumor progression or death. Disease-specific survival (DSS) provides information on the number of deaths due to a specific disease.

SPSS software (ver. 20.0; SPSS Inc., Chicago, IL, USA) was used for the statistical analyses. For baseline data analysis, the chi-square or Fisher’s exact test was used for categorical variables and one-way analysis of variance (ANOVA) for continuous variables. Kaplan–Meier survival curves were drawn and the log-rank test was used for comparisons. Multiple parameters were analyzed by multivariate Cox proportional hazards regression. *P*<0.05 was considered statistically significant in all tests.

## Results

### Demographic Characteristics

Of the 106 patients, 30 (28.3%) were classified as UrAC, 40 (37.7%) as primary BAC, and 36 (34.0%) as UCGD (**Table 1**). The mean age at diagnosis was 59.19 ± 13.82 years. Patients with UrAC were younger (51.87 ± 15.25 years) than those with primary BAC(60.50 ± 12.56 years) and UCGD (63.83 ± 11.60 years) (*P*<0.001), while no group difference was found in the gender distribution. In total, 46.7% of patients with UrAC were younger than 50 years, compared to only 22.5% and 5.6% of those in the primary BAC and UCGD groups, respectively (*P*=0.*006*). Patients with UrAC were more likely to be T3–4 stage (70.0 *vs*. 40.0% *vs*. 44.4%; *P*<0.001). Similarly, a higher proportion of UrAC group patients were stage III–IV (66.7%) compared to the primary BAC (40.0%) and UCGD (47.2%) groups (*P*=0.130), although the difference was not statistically significant. Interestingly, when only the UrAC and primary BAC groups were compared, the difference was significant (*P*<0.0036). Moreover, poor differentiation was more prevalent in the primary BAC than UrAC and UCGD groups (57.4 *vs*. 18.5, 24.1%; *P*<0.001). Regarding management, more than half of the UrAC patients underwent partial cystectomy (73.3% on diagnosis compared with 30.0% and 2.8% of the primary BAC and UCGD patients, respectively (*P*<0.001). In the UrAC and primary BAC groups, the rates of histological subtypes were as follows: 43.3% *vs*. 15.0% for mucinous adenocarcinoma, 46.7% *vs*. 67.5% for NOS, and 10% *vs*. 17.5% for others (*P*=0.030). **Table 1** summarizes the demographic and tumor characteristics.

**Table 1:** Patient’s baseline characteristics.

	*UrAC*	*Primary BAC*	*UCGD*	*χ2 /F value*	*P value*
**No.**	30	40	36	
**Sex**					
man	22 (73.3%)	26 (65.0%)	29 (80.6%)	2.317	0.314
woman	8 (26.7%)	14 (35.0%)	7 (19.4%)		
**Age (median ± SD, y)**	51.87±15.25	60.50±12.56	63.83±11.60	7.179	0.001
<50	14 (46.7%)	9 (22.5%)	2 (5.6%)	18.290	0.006
50-59	5 (16.7%)	10 (25.0%)	13 (36.1%)
60-69	9 (30.0%)	11 (27.5%)	11 (30.6%)
≥70	2 (6.7%)	10 (25.0%)	10 (27.8%)
**Tumor size (cm)**	3.42±1.60	2.73±1.30	3.54±1.69	3.037	0.052
≤4	21 (70.0%)	35 (87.5%)	27 (75.0%)	3.440	0.179
>4	9 (30.0%)	5 (12.5%)	9 (25.0%)
**T stage**					
≤T1	3 (10.0%)	16 (40.0%)	10 (27.8%)	22.290	0.001
T2	6 (20.0%)	8 (20.0%)	10 (27.8%)
T3	21 (70.0%)	13 (32.5%)	9 (25.0%)
T4	0 (0.0%)	3 (7.5%)	7 (19.4%)
**N stage**					
N0	28 (81.5%)	37 (92.5%)	30 (83.3%)	7.740	0.258
N1	1 (3.3%)	0 (0.0%)	3 (8.3%)
N2	1 (3.3%)	0 (0.0%)	2 (5.6%)
N3	0 (0.0%)	3 (7.5%)	1 (2.8%)
**Stage (AJCC 8^th^)**					
I	4 (13.3%)	16 (40%)	10 (27.8%)	7.109	0.130
II	6 (20.0%)	8 (20%)	9 (25.0%)
III+IV	20 (66.7%)	16 (40%)	17 (47.2%)
**Histology**					
Mucinous	13 (43.3%)	6 (15.0%)	–	7.016	0.030
NOS	14 (46.7%)	27 (67.5%)	–
others	3 (10.0%)	7 (17.5%)	–
**Grade**					
Poorly differentiated	10 (33.3%)	31 (77.5%)	13 (36.1%)	20.532	0.001
Moderately differentiated	20 (66.7%)	8 (20%)	21 (58.3%)
Well differentiated	0 (0.0%)	1 (2.5%)	2 (5.6%)
**Management**					
Transurethral resection	3 (10.0%)	11 (27.5%)	11 (30.6%)	41.893	0.001
Partial cystectomy	22 (73.3%)	12 (30.0%)	1 (2.8%)
Cystectomy	4 (13.3%)	13 (32.5%)	23 (63.9%)
None/other	1 (3.3%)	4 (10.0%)	1 (2.8%)
**Chemotherapy**					
No	21 (70.0%)	29 (72.5%)	23 (63.9%)	0.680	0.712
Yes	9 (30.0%)	11 (27.5%)	13 (36.1%)
**Concomitant disease**					
No	15 (50%)	21 (52.5%)	16 (44.4%)	5.571	0.850
Diabetes	2 (6.7%)	5 (12.5%)	5 (13.9%)
Hypertension	9 (30.0%)	8 (20.0%)	12 (33.3%)
Coronary heart disease	4 (13.3%)	4 (10.0%)	5 (13.9%)
Cerebral infarction	1 (3.3%)	1 (2.5%)	2 (5.6%)
Other major diseases	4 (13.3%)	13 (32.5%)	8 (22.2%)
**KI-67 (%)**					
≤50	6 (20.0%)	6 (15.0%)	9 (25.0%)	2.704	0.609
>50	10 (33.3%)	9 (22.5%)	9 (25.0%)
undertermined	14 (46.7%)	25 (62.5%)	18 (50.0%)
**aCCI scores**	3.50±1.68	4.23±1.67	4.42±1.52	2.850	0.062
0-2	12 (40.0%)	7 (17.5%)	2 (5.6%)	13.054	0.011
3-5	15 (50.0%)	24 (60.%)	26 (72.2%)
6-8	3 (10.0%)	9 (22.5%)	8 (22.2%)
**BMI scores (kg/m^2^)**	24.66 ±2.90	23.71 ±3.47	23.97± 3.58	0.679	0.510
<23	8 (26.7%)	17 (42.5%)	13 (36.1%)	2.559	0.634
23∼27.5	16 (53.3%)	18 (45.0%)	19 (52.8%)
≥27.5	6 (20.0%)	5 (12.5%)	4 (11.1%)

NOS, not otherwise specified; aCCI, age-adjusted Charlson Comorbidity Index; BMI, body mass index; UrAC, urachal adenocarcinoma; BAC, bladder adenocarcinoma; UCGD, urothelial carcinoma with glandular differentiation.

### Survival Analyses

**Figure 1:**
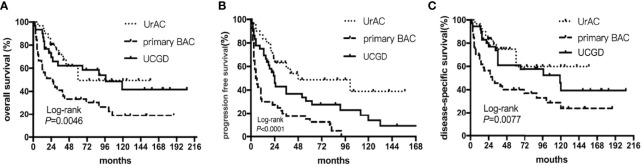
Kaplan–Meier curves of patients stratified according to tumor type: UrAC *vs*. primary BAC *vs*. UCGD. **(A)** overall survival (*P*=0.0046, log-rank test). **(B)** progression free survival (*P*<0.0001, log-rank test). **(C)** disease-specific survival (*P*=0.0077, log-rank test).

The date of the last follow-up was August 1, 2021. The median follow-up time was 36 (13–88) months. The 5-year OS rates were 50.8%, 34.9%, and 48.4% for the UrAC, primary BAC, and UCGD groups, respectively. **Table 2** shows the 1-, 3-, and 5-year survival rates of the three groups. The unadjusted Kaplan–Meier curves showed that the OS and DSS rates of the primary BAC group were poorer than those of the UrAC and UCGD groups (*P*<0.05, **Figures 1A, C**). The PFS rate was highest for the UrAC group and lowest for the primary BAC group (*P*<0.001, **Figure 1B**). Regarding BAC, patients with mucinous-type adenocarcinoma tended to have better OS and PFS than those with other histological types (*P*<0.05, log-rank test) (**Supplementary Figure 1**).Twelve other factors that may affect survival were analyzed by the log-rank test (sex, age, tumor size, T stage, N stage, total stage, grade, management, chemotherapy, ki67, aCCI score, and BMI). Of these factors, six significantly (*P*<0.05) influenced OS separately: age, T stage, N stage, grade, management, and aCCI score. In addition, five factors significantly (*P*<0.05) influenced PFS and DSS separately: age, T stage, N stage, grade, and aCCI score. In our multivariate adjusted Cox regression model, tumor type (P=0.002), T stage (P=0.034), and aCCI score (P=0.005) predicted OS and DSS **(** **Table 3** and **Table S2**). Tumor type (P=0.011), grade (P=0.000) and aCCI (P=0.002) scores were predictors of PFS **(** **Table S1**).

**Table 2:** Survival comparison of the three groups.

	1 year survival			3 year survival			5 year survival		
	OS	PFS	DSS	OS	PFS	DSS	OS	PFS	DSS
UrAC	93.3%	83.3%	96.7%	69.7%	58.6%	74.8%	50.8%	48.7%	59.8%
primary BAC	63.2%	30%	69.8%	43.4%	17.5%	45.7%	34.9%	12.5%	40.1%
UCGD	87.7%	75%	94.4%	62.5%	42.9%	73.7%	48.4%	30.7%	60.9%

UrAC, urachal adenocarcinoma; BAC, bladder adenocarcinoma; UCGD, urothelial carcinoma with glandular differentiation; OS, overall survival; PFS, progression free survival; DSS, disease-specific survival.

**Table 3:** Association of factors with overall survival on multivariate Cox proportional hazards regression analysis.

Characteristic	Multivariate analysis	
	Hazards ratio (95% CI)	P value
**type**		
UrAC	reference	
primary BAC	2.495 (1.238-5.029)	0.011
UCGD	0.959 (0.417-2.203)	0.921
**Age categories(y)**		
<50	reference	
50-59	0.489 (0.123-1.949)	0.311
60-69	0.378 (0.093-1.532)	0.173
≥70	0.688 (0.167-2.838)	0.605
**T stage**		
T1	reference	
T2	2.672 (1.020-7.003)	0.046
T3	4.300 (1.803-10.259)	0.001
T4	2.081 (0.734-5.906)	0.168
**N stage**		
N0	reference	
N1	0.538 (0.120-2.407)	0.417
N2	1.261 (0.269-5.917)	0.769
N3	2.451 (0.617-9.741)	0.203
**Grade**		
Poorly differentiated	reference	
Moderately differentiated	0.542 (0.289-1.017)	0.057
Well differentiated	0.472 (0.062-3.578)	0.468
**Management**		
Transurethral resection	reference	
Partial cystectomy	0.572 (0.222-1.476)	0.248
Cystectomy	0.884 (0.372-2.100)	0.779
None/other	2.274 (0.733-7.061)	0.155
**aCCI scores**		
0-2	reference	
3-5	2.489 (1.067-5.806)	0.035
6-8	5.265 (2.039-13.595)	0.001

aCCI, age-adjusted Charlson Comorbidity Index; UrAC, urachal adenocarcinoma; BAC, bladder adenocarcinoma; UCGD, urothelial carcinoma with glandular differentiation.

## Discussion

UrAC, primary BAC and UCGD are rare pathological types of bladder cancer that have been relatively understudied due to the small numbers of cases. There have been five comparative systematic prognostic studies of UrAC and primary BAC, none of which examined a Chinese population (8–11, 16). And the prognostic difference between BAC and UCGD, two clinically confused diseases, is rarely reported. Therefore, this study analyzed cases from 2005 to 2019, seen at a single center in China, to compare the prognosis and prognostic factors of UrAC, primary BAC, and UCGD.

In our series, we found significant differences in clinical and neoplastic features among the UrAC, primary BAC, and UCGD groups. The median age of the UrAC group was about 10 years lower (52 years) compared with the primary BAC and UCGD groups (61 and 64 years, respectively). There was an overwhelming male predominance in all three groups. However, the previously reported significantly higher proportion of men with UrAC versus the other types was not seen, which we believe was related to the small sample size. Interestingly, poorly differentiated tumors were less common among the UrAC patients, although they had more advanced T stages. Combined with previous studies, we believe that high stage and high differentiation are clinicopathological features of UrAC in particular. Partial cystectomy was the most common surgical method for UrAC, while total cystectomy was the most common for UCGD (17, 18). However, almost equal numbers of primary BAC patients underwent transurethral resection, partial cystectomy, and total cystectomy as the first operation. We believe that this is attributable to surgeon preferences. Many factors play a role in management decisions, such as patient preference, tumor location, degree of differentiation, whether the tumor is isolated, and comorbidities. BAC can perform various phenotypes: mucinous/colloid, enteric/colonic, signet-ring cell, clear cell, hepatoid, mixed and adenocarcinoma not otherwise specified (NOS; if without a specific glandular growth pattern) (12). Mucinous adenocarcinoma is considered a less aggressive histological subtype (19) and was more common in UrAC than primary BAC patients in this study, consistent with previous results.

The survival advantage of UrAC is controversial. Grignonet et al. (11) reported a 5-year OS of 61% for UrAC and 31% for primary BAC. Wright et al. (8) found that the 5-year OS of UrAC patients was higher than that of primary BAC patients. Anderstromet et al. (19) published similar results, a survival advantage for UrAC, although this was not statistically significant. By contrast, Wilson et al. reported a 3-year cancer-specific survival rate of 31% for patients with UrAC and 48% for those with primary BAC (20). The reported 5-year survival rates are 27–61% and 11–55% for UrAC and primary BAC, respectively (5, 21–23). In our series, we found that patients with UrAC had a longer median survival time and lower risk of death compared with patients with primary BAC.

Bladder cancer is a heterogeneous entity characterized by a wide range of morphologies and clinical processes. Due to the ability of urothelial cells to differentiate polytropically and heterotrophically, there are many heterotrophic subtypes. In 2016, the WHO distinguished 12 subtypes of UC, including those with squamous differentiation and glandular differentiation (24). The WHO defined UCGD as a mixed tumor with glandular and urothelial differentiation, because its clinical manifestations and pathology are similar to many other bladder lesions, including BAC (which is prone to misdiagnosis and mismanagement) (25). Reports on the prognosis of UC and UCGD indicate that patients with glandular differentiation have more obvious tumor invasion and relatively poor postoperative recovery, although in other reports the difference in prognosis was not significant (26–29).

And several studies have reported the prognoses of BAC and UC. One study showed that patients with muscle-infiltrating BAC had similar survival rates to those with muscle-infiltrating UC (30). A SEER database analysis found that the 5-year cancer specific survival rate was 56.6% for patients with primary BAC and 61% for those with UC (27). To conclude, many studies have reported differences between UCGD and UC, UC and BAC. However, the difference in prognosis between BAC and UCGD has rarely been reported. Our study fills in the data.

Our study had many shortcomings. First, the sample size was relatively small, which is a common problem due to the rarity of the diseases. Second, this was a retrospective study; the rarity of the diseases makes prospective studies almost impossible. In addition, many patients did not undergo lymph node dissection, so the N stage results can only be used as a reference. Despite these shortcomings, this study provides valuable data for patient counseling, treatment planning, and prognostic predictions. Although few cases were included in this study compared to the SEER database, our data are reliable and detailed. As one of the largest urology treatment centers in China, we believe that this analysis of 15 years of follow-up data is highly meaningful. Larger, multicenter studies should nevertheless be performed to validate our results.

## Conclusion

Our study found that UrAC, primary BAC, and UCGD differed in pathological and clinical features. Patients with UrAC were the most likely to be stage T3–4 and the primary BAC patients had the highest rate of poorly differentiated tumors. Patients with mucinous adenocarcinoma tended to have better OS and PFS. This analysis of UrAC, primary BAC, and UCGD indicated a worse prognosis for cases with primary BAC. Attempts should be made to diagnose these aggressive tumors early, since patients in whom tumors are detected early appear to survive longer.

## Data Availability Statement

The original contributions presented in the study are included in the article/**Supplementary Material**. Further inquiries can be directed to the corresponding authors.

## Ethics Statement

The studies involving human participants were reviewed and approved by People’s Liberation Army General Hospital. Written informed consent for participation was not required for this study in accordance with the national legislation and the institutional requirements.

## Author Contributions

Conception and design: XL and TW. Acquisition of data: ZL and HF. Analysis and interpretation of data: TW and XL. Writing, review, and/or revision of the manuscript: TW, YY, XH, XZ, BC, and ZL. Administrative, technical, or material support: JL and XM. Study supervision: XL. All authors contributed to the article and approved the submitted version.

## Funding

The present work was financially supported by the National Natural Science Foundation of China (No. 81900718 and No.81970665) and Chinese Postdoctoral Science Foundation (No.BSH47933-JD).

## Conflict of Interest

The authors declare that the research was conducted in the absence of any commercial or financial relationships that could be construed as a potential conflict of interest.

## Publisher’s Note

All claims expressed in this article are solely those of the authors and do not necessarily represent those of their affiliated organizations, or those of the publisher, the editors and the reviewers. Any product that may be evaluated in this article, or claim that may be made by its manufacturer, is not guaranteed or endorsed by the publisher.

## Supplementary Material

Supplementary Figure 1 Kaplan–Meier curves of the BAC patients stratified according to tumor type: mucinous *vs*. NOS *vs*. Others.**(A)** overall survival (*P*=0.0050, log-rank test). **(B)** progression free survival (*P*=0.0245, log-rank test). **(C)** disease-specific survival (*P*=0.0924, log-rank test).

Supplementary Figure 2 Histological features of three types of tumors. **(A)** urachal adenocarcinomas. **(B)** primary bladder adenocarcinomas. **(C)** urothelial carcinoma with glandular differentiation.
