# Supplementary figures and images for: Survival of Patients With UrAC and Primary BAC and Urothelial Carcinoma With Glandular Differentiation
Source: Front Oncol. 2022 May 12;12:860133. doi: 10.3389/fonc.2022.860133 (PMC9133414; doi:10.3389/fonc.2022.860133)

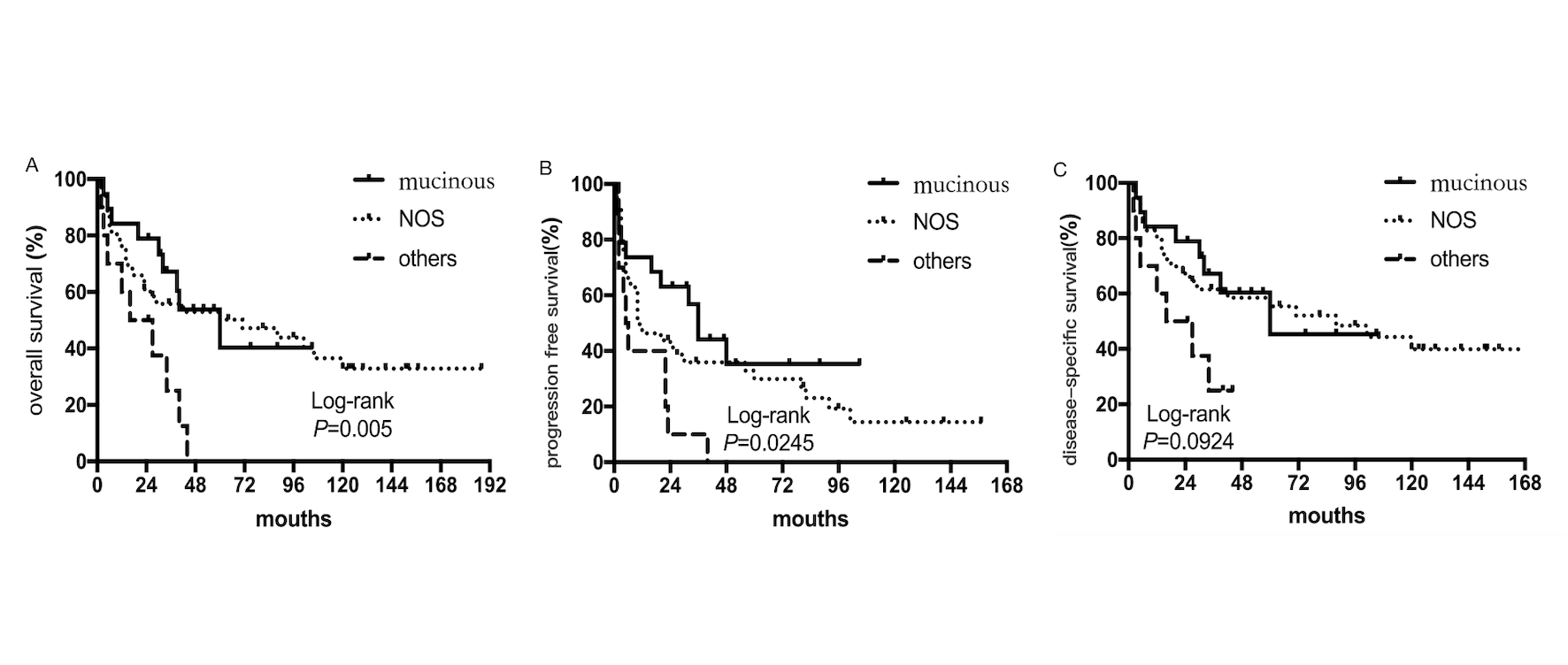

Supplement: Supplementary Figure 1 — Kaplan–Meier curves of the BAC patients stratified according to tumor type: mucinous vs. NOS vs. Others.(A) overall survival (P=0.0050, log-rank test). (B) progression free survival (P=0.0245, log-rank test). (C) disease-specific survival (P=0.0924, log-rank test). [file Image_1.jpeg]

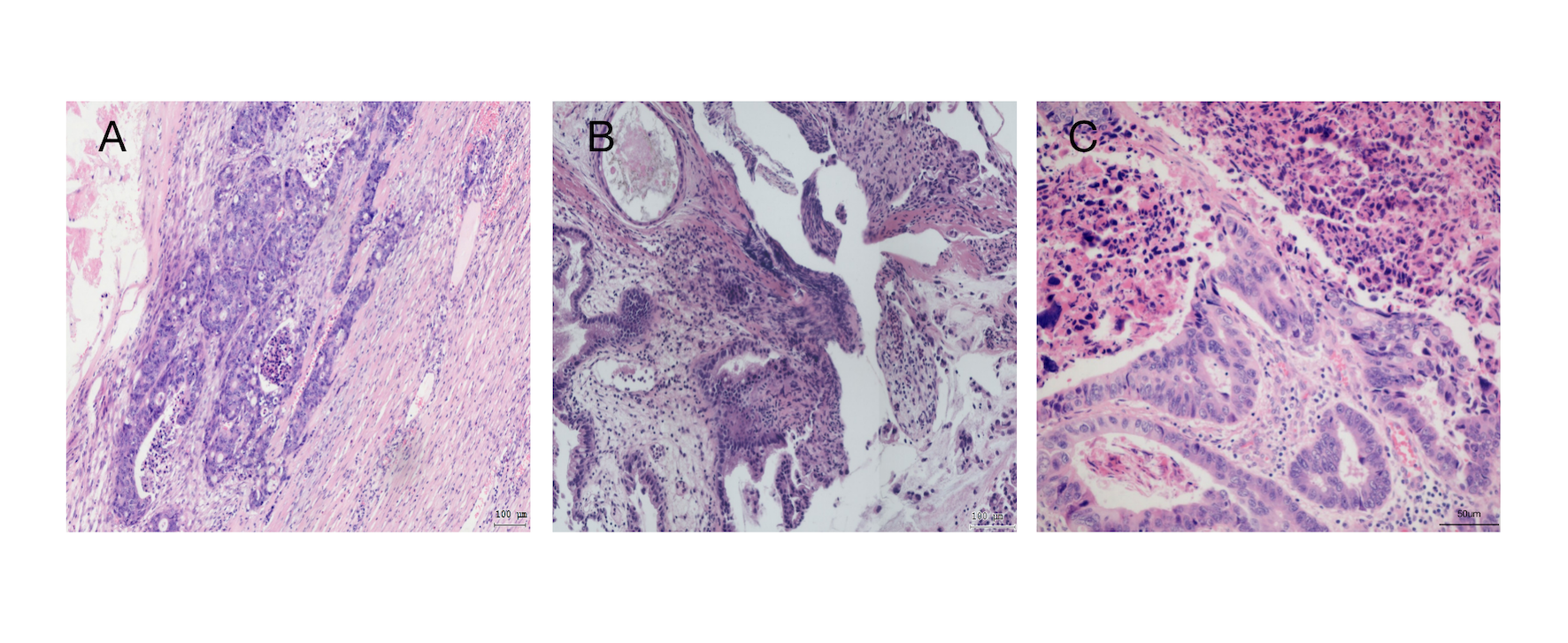

Supplement: Supplementary Figure 2 — Histological features of three types of tumors. (A) urachal adenocarcinomas. (B) primary bladder adenocarcinomas. (C) urothelial carcinoma with glandular differentiation. [file Image_2.jpeg]
