# Supplementary material for: Survival of Patients With UrAC and Primary BAC and Urothelial Carcinoma With Glandular Differentiation
Source: Front Oncol. 2022 May 12;12:860133. doi: 10.3389/fonc.2022.860133 (PMC9133414; doi:10.3389/fonc.2022.860133)
Supplement: Supplementary file 3 [file DataSheet_1.docx]

Table S1. Association of factors with progression free survival on multivariate Cox proportional hazards regression analysis.

| ***characteristic*** | ***Multivariate analysis*** | |
| --- | --- | --- |
|  | ***Hazards ratio(95% CI)*** | ***P value*** |
| **type** |  |  |
| UrAC | reference |  |
| primary BAC | 2.529（1.305-4.900） | 0.006 |
| UCGD | 1.339（0.692-2.590） | 0.386 |
| **Age categories(y)** |  |  |
| <50 | reference |  |
| 50-59 | 1.261(0.404-3.936) | 0.690 |
| 60-69 | 1.011(0.322-3.180) | 0.984 |
| ≥70 | 1.590(0.450-5.615) | 0.471 |
| **T stage** |  |  |
| T1 | reference |  |
| T2 | 0.909(0.466-1.774) | 0.780 |
| T3 | 1.604(0.884-2.911) | 0.120 |
| T4 | 1.304(0.562-3.028) | 0.537 |
| **N stage** |  |  |
| N0 | reference |  |
| N1 | 0.514(0.121-2.177) | 0.366 |
| N2 | 2.979(0.875-10.145) | 0.081 |
| N3 | 2.739(0.816-9.191) | 0.103 |
| **Grade** |  |  |
| Poorly differentiated | reference |  |
| Moderately differentiated | 0.371(0.216-0.635) | 0.000 |
| Well differentiated | 0.157(0.021-1.157) | 0.069 |
| **aCCI scores** |  |  |
| 0-2 | reference |  |
| 3-5 | 1.914(0.964-3.803) | 0.064 |
| 6-8 | 3.966(1.786-8.806) | 0.001 |

aCCI = age-adjusted Charlson Comorbidity Index.

UrAC = urachal adenocarcinoma.

BAC = bladder adenocarcinoma.

UCGD = urothelial carcinoma with glandular differentiation.

Table S2. Association of factors with disease-specific survival on multivariate Cox proportional hazards regression analysis.

| ***characteristic*** | ***Multivariate analysis*** | |
| --- | --- | --- |
|  | ***Hazards ratio(95% CI)*** | ***P value*** |
| **type** |  |  |
| UrAC | reference |  |
| primary BAC | 3.500(1.610-7.609) | 0.002 |
| UCGD | 1.691(0.720-3.968) | 0.228 |
| **Age categories(y)** |  |  |
| <50 | reference |  |
| 50-59 | 2.221(0.248-19.895) | 0.476 |
| 60-69 | 1.828(0.209-16.008) | 0.586 |
| ≥70 | 1.835(0.215-15.659) | 0.579 |
| **T stage** |  |  |
| T1 | reference |  |
| T2 | 1.503(0.664-3.401) | 0.328 |
| T3 | 2.496(1.234-5.046) | 0.011 |
| T4 | 1.853(0.673-5.099) | 0.232 |
| **N stage** |  |  |
| N0 | reference |  |
| N1 | 0.757(0.167-3.434) | 0.719 |
| N2 | 0.678(0.086-5.349) | 0.712 |
| N3 | 2.869(0.770-10.691) | 0.116 |
| **Grade** |  |  |
| Poorly differentiated | reference |  |
| Moderately differentiated | 0.525(0.266-1.038) | 0.064 |
| Well differentiated | 0.561(0.073-4.288) | 0.578 |
| **aCCI scores** |  |  |
| 0-2 | reference |  |
| 3-5 | 2.176(0.931-5.085) | 0.073 |
| 6-8 | 2.944(1.069-8.111) | 0.037 |

aCCI = age-adjusted Charlson Comorbidity Index.

UrAC = urachal adenocarcinoma.

BAC = bladder adenocarcinoma.

UCGD = urothelial carcinoma with glandular differentiation.
